# Polyelectrolyte-Nanoplatelet Complexation: Is It Possible to Predict the State Diagram?

**DOI:** 10.3390/ijms20246217

**Published:** 2019-12-10

**Authors:** Maria Jansson, Marie Skepö

**Affiliations:** 1 Theoretical Chemistry, Lund University, P.O. Box 124, SE-221 00 Lund, Sweden; 2 LINXS—Lund Institute of Advanced Neutron and X-ray Science, Scheelevägen 19, SE-223 70 Lund, Sweden

**Keywords:** polyelectrolyte, nanoplatelet, complexation, molecular dynamics simulations, charge stoichiometry, chain length, linear charge density, chain flexibility

## Abstract

The addition of polyelectrolytes (PEs) to suspensions of charged colloids, such as nanoplatelets (NPs), is of great interest due to their specific feature of being either a stabilizing or a destabilizing agent. Here, the complexation between a PE and oppositely charged NPs is studied utilizing coarse-grained molecular dynamics simulations based on the continuum model. The complex formation is evaluated with respect to the stoichiometric charge-ratio within the system, as well as by the alternation of the chain properties. It is found that the formed complexes can possess either an extended or a compact shape. Moreover, it is observed that the chain can become overcharged by the oppositely charged NPs. With an increase in chain length, or a decrease in chain flexibility, the complex obtains a more extended shape, where the NPs are less tightly bound to the PE. The latter is also true when reducing the total charge of the chain by varying the linear charge density, whereas in this case, the chain contracts. With our coarse-grained model and molecular dynamics simulations, we are able to predict the composition and the shape of the formed complex and how it is affected by the characteristics of the chain. The take-home message is that the complexation between PEs and NPs results in a versatile and rich state diagram, which indeed is difficult to predict, and dependent on the properties of the chain and the model used. Thus, we propose that the present model can be a useful tool to achieve an understanding of the PE-NPs complexation, a system commonly used in industrial and in technological processes.

## 1. Introduction

The complexation between polyelectrolytes (PEs) and oppositely charged colloidal particles is of great interest for various industrial and technological processes [1,2]. PEs are highly soluble in water due to their charged nature and adsorb strongly to oppositely charged particles [3,4,5,6,7,8]. There exists a large variety of natural and synthetic PEs with different properties, which are used for a wide range of applications [9,10]. One specific feature is that a suspension of charged colloids can be either stabilized or destabilized with the addition of an oppositely charged PE [11,12,13]. Nanoplatelets (NPs), such as clays, have both an anisotropic shape and charge, where the lateral dimensions can be several orders of magnitude larger than the thickness [14]. The surface, i.e., the face of the NP, usually holds a high negative charge, while the rim can either be negative, neutral, or positive, depending on the pH of the solution [15,16]. The complex formation of PEs and NPs depends strongly on the properties of the macromolecules and a variation of those results in a state diagram that contains a rich and versatile variety of structures. The effect of the PEs’ properties has been studied previously for PE-macroion complexation by utilizing canonical Monte Carlo simulations [17,18]. The authors investigated the complex formation between one PE and an addition of oppositely charged spherical macroions with respect to the linear charge density, length, and flexibility. It was found that the capacity to complex macroions decreased with a reduction in the linear charge density or in the chain length, where the former gave rise to a looser complex and the latter to the opposite. Further, it was observed that with a decrease in the flexibility of the PE, the ability to overcharge the complex increased, where the macroions in the complex became more linearly arranged.

Recently, the intercalation of a highly charged unstructured peptide within the layered structure of the synthetic clay LAPONITE^®^[19] was studied [20]. It was found that complexes consisting of stacked NPs, also denoted tactoids, were formed as a function of the peptide concentration. Above a matched charge-ratio between the peptide and the clay, the growth of the tactoids was inhibited, and a tactoidal dissolution was observed, caused by the excess of chains and, thus, overcharging of the NPs by the former. In the present work, the complexation between a positively charged PE and anisotropically charged NPs, where the latter consists of a negatively charged surface and a positively charged rim, is studied with coarse-grained molecular dynamics (MD) simulations based on the continuum model. The importance of the stoichiometric charge-ratio on the formed complex, as well as on the inherent PE properties is investigated. Furthermore, alternation of the PEs properties is performed in order to determine the preferred shape and the composition of the formed complex, as well as the conformation of the PE. By using MD simulations, we show that the complex formation depends strongly on the PE properties and that the complex adopts a variety of different structures.

## 2. Theoretical Section

### 2.1. The Coarse-Grained Model

**Figure 1:**
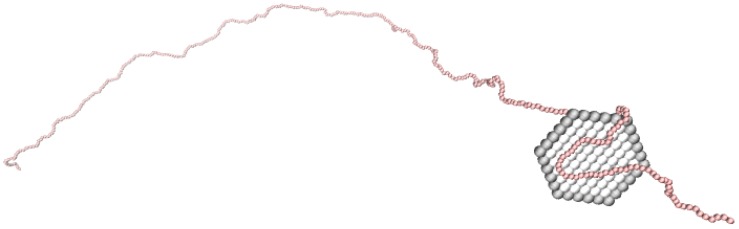
Schematic representation of the coarse-grained model for a system consisting of one nanoplatelet (NP) and one polyelectrolyte (PE) with $N_{b}=440$. The face (light grey) and the rim (dark grey) of the NP have a total charge of $Z_{\mathrm{face}}=-61$ and $Z_{\mathrm{rim}}=+6$, respectively, and the PE beads (red) each have a charge of $Z_{b}=+1$. The NP is modeled to mimic a clay platelet.

In the simulation model, the NP is modeled as a finite hexagonal monolayer of connected coarse-grained spheres, defined as sites. The thickness and the diameter of the NP were 1 and 11 nm, respectively, where the diameter was smaller than the experimental one in order to reduce the computational cost. The model of the NP was mimicking the properties of clay [21]. The unit charges were located at the center of each site to obtain a homogeneous charge distribution over the NP. Charge anisotropy was implemented by adding both face and rim elementary charges with $Z_{\mathrm{face}}=-61$, and $Z_{\mathrm{rim}}=+6$; hence, the total net elementary charge of the NP was $Z_{p}=-55$. The PE was modeled as a positively charged chain of connected coarse-grained beads, each with a elementary charge of $Z_{b}=+1$. A schematic representation of the NP, i.e., the clay platelet, and the PE is shown in Figure 1.

The total NP and PE charges were neutralized by monovalent counterions, which were added explicitly, and modeled as freely moving charges. The dimensionless stoichiometric charge-ratio in the system, and the complexed charge-ratio, will be used throughout to describe the system and the complex with respect to under- and overcharging. The former is defined as the absolute value of the NPs’ total net negative charge divided by the number of positive PE charges, i.e.,

(1) $$\beta =|\frac{N_{p}\cdot Z_{p}}{N_{b}\cdot Z_{b}}|\;.$$

$N_{p}$ is the number of NPs, which was varied from one to 16, in order to obtain a stoichiometric charge-ratio of β= 0.13–2.0, and $N_{b}$ is the number of beads within the PE chain. The complexed charge-ratio is defined as the ratio between the complexed NPs’ total net negative charge and the total positive charges of the PE, i.e.,

(2) $$\beta^{c}=|\frac{N_{p}^{c}\cdot Z_{p}}{N_{b}\cdot Z_{b}}|\;,$$

where $N_{p}^{c}$ is the number of NPs complexed to the PE. Hence, the case β<1.0 represents a system with more PE charges than NP charges, β=1.0 an equal amount of PE and NP charges, and β>1.0 and excess of NP charges. Moreover, $\beta^{c}<1.0$ refers to an undercharged complex, $\beta^{c}=1.0$ to a neutral complex, and $\beta^{c}<1.0$ to an overcharged complex.

To investigate how the complexation between the PE and the NPs depends on the properties of the former, the effect of the chain length, the total charge through the linear charge density, and the flexibility were studied. For the chain length, the number of beads was varied between 440 and 800, with an addition of eight NPs. Regarding the total charge and the flexibility, the PE with $N_{b}=$ 440 was used with varied linear charge density or angular potential. Furthermore, the effect of the NPs’ rim was studied by setting the rim charge in the model to zero for the PE with $N_{b}=488$. The reader should notice that a PE with a length of 440 and 488 either matches the total net negative charge or the negative face charges of a system where eight NPs have been added. The specifications of the systems evaluated in this study are compiled in Table 1.

In the simulations, the solvent was treated implicitly as a dielectric continuum with the dielectric constant of $\epsilon_{r}=78.3$. All the interactions in the system were assumed to be pairwise additive, and the electrostatic pair potential is defined as:

(3) $$u_{ij}^{\mathrm{EL}}\left(r_{ij}\right)=\frac{e^{2}z_{i}z_{j}}{4\pi \epsilon_{0}\epsilon_{r}r_{ij}}\;,$$

where *e* is the elementary charge, $z_{i}$ is the valency of beads/sites within the PE/NP *i*, $\epsilon_{0}$ is the permittivity of the vacuum, and $r_{ij}$ is the center-to-center distance between beads/sites *i* and *j*. In addition, the beads/sites also interact via a strictly repulsive, truncated, and shifted Lennard–Jones (TLJ) potential, defined as:

(4) $$u_{ij}^{\mathrm{TLJ}}\left(r_{ij}\right)=\left\{\begin{array}{cc} \epsilon_{\mathrm{TLJ}}\left[{\left(\frac{\sigma_{ij}}{r_{ij}}\right)}^{12}-2{\left(\frac{\sigma_{ij}}{r_{ij}}\right)}^{6}+1\right], & \;\mathrm{if}\;r_{ij}<\sigma_{ij}\\ 0, & \;\mathrm{otherwise} \end{array}\right.\;.$$

The strength of the short range potential was set to $\beta \epsilon_{\mathrm{TLJ}}=1$, where β=1/$k_{B}T$, $k_{B}$ is the Boltzmann constant, and *T* = 298 K. $\sigma_{ij}=\left(\sigma_{i}+\sigma_{j}\right)/2$, and $\sigma_{i}$ is the diameter of species *i*, where $\sigma_{ion}=\sigma_{b}=0.4$ nm and $\sigma_{site}=1.0$ nm. Thus, the total potential between all pairs is defined as:

(5) $$u_{ij}\left(r_{ij}\right)=u_{ij}^{\mathrm{TLJ}}\left(r_{ij}\right)+u_{ij}^{\mathrm{EL}}\left(r_{ij}\right)\;.$$

Furthermore, all the adjacent beads/sites within the PE/NP are connected by a harmonic bond potential:

(6) $$u_{b}\left(r_{ij}\right)=\frac{1}{2}k^{b}{\left(r_{ij}-b\right)}^{2}\;.$$

$k^{b}$ is the force constant, and *b* is the equilibrium bond length, where b= 0.5 and 1.0 nm for the PE and the NP, respectively. The flexibilities of the PE/NP were constrained by a harmonic angular potential between triplets of beads/sites:

(7) $$u_{}\left(\theta_{ijk}\right)=\frac{1}{2}k^{\theta }{\left(\theta_{ijk}-\theta^{0}\right)}^{2}\;,$$

where $k^{\theta }$ is the force constant, $\theta_{ijk}$ is the bond angle between beads/sites *i*, *j*, and *k*, and $\theta^{0}=\pi$.

### 2.2. Molecular Dynamics Simulations

**Figure 2:**
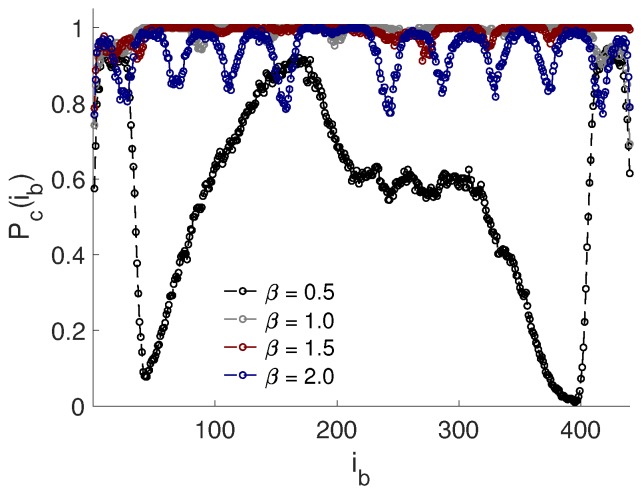
Complexation probability, $P_{c}\left(i_{b}\right)$, as a function of the bead number, $i_{b}$, for $N_{b}=440$.

The MD simulations were performed with the software package GROMACS (Version 5.0.4) [22]. A cubic simulation box with three-dimensional periodic boundary conditions was used, with a box length of 100 nm, corresponding to a volume fraction of ∼0.005–0.08%, and ∼0.002–0.003% for 1–16 NPs and for the chain length of $N_{b}=$ 440–800, respectively. Due to the very low volume fraction of the added particles, the dielectric constant of the solution was not expected to be affected. The time step was set to 10 fs, with a total of $10^{7}$ steps. Newton’s equation of motion of freely moving species, i.e., the NPs, the PE, and the counterions, was integrated using the leap-frog algorithm. To account for the long range electrostatic contribution, fast particle-mesh Ewald summation was utilized with a 6 nm real-space Coulomb cutoff and a Fourier spacing equal to 0.6 nm. The thermostat was v-rescale, a temperature coupling using velocity rescaling with a stochastic term, and the solvent was treated implicitly with the dielectric constant of $\epsilon_{r}=78.3$. For an in-depth description of the input parameters, the reader is referred to the user manual [22]. The convergence of the system was evaluated from the complexation probability ($P_{c}\left(i_{b}\right)$) function. This provides information about the probability that the bead, $i_{b}$, in the PE is complexed with either one or several NPs and, thus, is a measure of the convergence of the sampling, i.e., both ends of the PE should experience the same environment. The threshold at which the bead was set to be complexed was determined from the bead-NP separation obtained from the radial distribution functions (data not shown) to <1.4 nm, i.e., when the separation between the center-of-mass of one PE bead and the NP did not exceed the distance $r=R_{\mathrm{site}}+R_{b}+0.7$ nm. The complexation probability function ranged from zero to one, where the lower boundary corresponded to the case where the bead was never complexed with the NP and the upper boundary corresponded to the case where the bead was always in a complex. The $P_{c}\left(i_{b}\right)$ for the reference system S.I is shown in Figure 2, and it is observed that all of the beads had a high complexation probability for β≥1.0. For β=0.5, the beads within the PE displayed both high and low complexation probabilities, which implies that not all of the beads were involved in the complex. However, simulations for replicates of the systems with different initial configurations were performed from which the same results were obtained, and hence, it can be concluded that they converged.

### 2.3. Structural Analysis

The structural information obtained from the simulations was analyzed by the NP-NP structure factor, defined as:

(8) $$S\left(q\right)=\langle \frac{1}{N}\sum_{i=1}^{N}\sum_{j=1}^{N}\frac{\sin(qr_{ij})}{qr_{ij}}\rangle \;,$$

where *N* is the total number of NP sites in the system. Assuming an isotropic scattering, the equation of the total structure factor can be rewritten as:

(9) $$S\left(q\right)=1+4\pi \frac{N}{V}\int_{0}^{\infty }\left(g\left(r\right)-1\right)r^{2}\frac{\sin(qr)}{qr}dr\;,$$

where *g(r)* is the radial distribution function between all the NP sites in the system. If *g(r)* is not approaching one at large separations due to the finite box length, a window function can be used to reduce the artifacts, according to:

(10) $$S_{w}\left(q\right)=1+4\pi \frac{N}{V}\int_{0}^{\infty }\left(g\left(r\right)-1\right)r^{2}\frac{\sin(qr)}{qr}\frac{\sin(\pi r/R_{c})}{\pi r/R_{c}}dr\;,$$

where $R_{c}$ is the maximum distance in *g(r)* [23].

## 3. Results and Discussion

### 3.1. Composition of the PE-NP Complex

**Figure 3:**
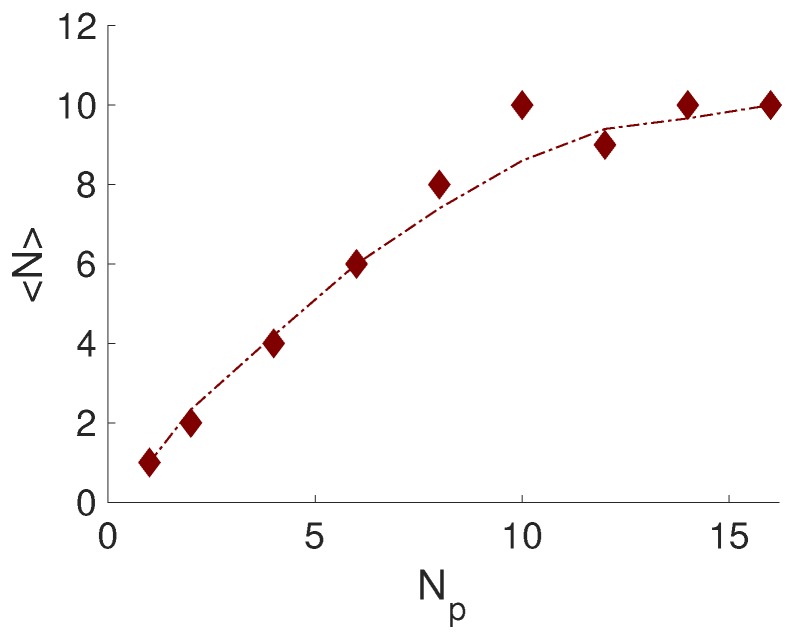
The average number of NPs complexed to the PE, 〈N〉, as a function of the number of NPs, $N_{p}$, added into the system. The dashed-dotted line is an implemented smooth function.

As a first step, the formation and composition of the formed complexes in the reference system, S.I, were considered. Figure 3 shows the average number of NPs complexed to the PE, 〈N〉, as a function of the number of NPs added into the system. It was observed that there was a linear increase in complexation to $N_{p}=10$ and that a plateau was reached. Moreover, from the complexed charge-ratio, $\beta^{c}$, it was found that the PE became overcharged by approximately 25%; see Figure S1 in the Supplementary Materials. This number was of the same order as was reported by Jonsson and Linse, when studying the complexation between a PE and oppositely charged spherical macroions [17,18].

**Figure 4:**
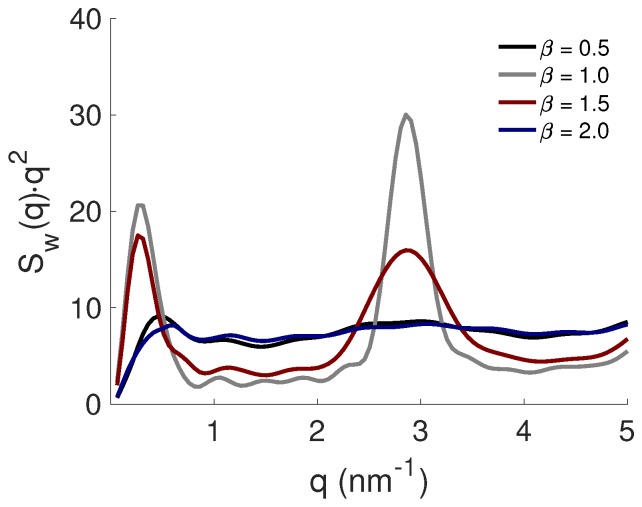
The NP-NP structure factors, $S_{w}\left(q\right)$, presented as Kratky plots for the PE with $N_{b}=440$.

The shape of the formed complexes was determined from the NP-NP structure factors, $S_{w}\left(q\right)$, presented as Kratky plots in Figure 4. Focusing on the low *q*-region, it was shown that when the PE was either under- or overcharged by the opposite charges of the NP, as for β= 0.5 and 2.0, the complex possessed a more extended shape, whereas when it was net neutral, or only slightly overcharged (β= 1.0–1.5), the complex obtained a compact shape. Furthermore, the peak intensity decreased with an increase in β, indicating that the most well ordered structure of the NPs was found at β=1.0. This was confirmed by the high *q*-region where both β= 1.0 and 1.5 exhibited Bragg peaks. At β > 1.5, the periodicity between the NPs decreased, suggesting a dissolution of the complex. The average distance, i.e., the *d*-spacing, between the mid-plane of two adjacent NPs was $d_{\mathrm{Bragg}}=$ 2.2 and 2.3 nm for β= 1.0 and 1.5, respectively, determined from the relation: $d_{\mathrm{Bragg}}=2\pi /q_{\max}$, where $q_{\max}$ is the position of the maximum intensity of the Bragg peak. These numbers were in correlation with the distances reported in the study regarding the intercalation of a highly charged peptide within LAPONITE^®^[19] layered clay minerals [20]. Moreover, the complexation of NPs induced by multivalent ions, such as calcium, was studied by a combination of MD simulations with experimental techniques [24]. The reported distance between the NPs was approximately ∼1.3 nm, which was almost half the size of the distance found in this study. Hence, this denotes that the distance between NPs was not affected by the PE length; instead, it depended on the type of added agent, i.e., a PE or an ion.

**Figure 5:**
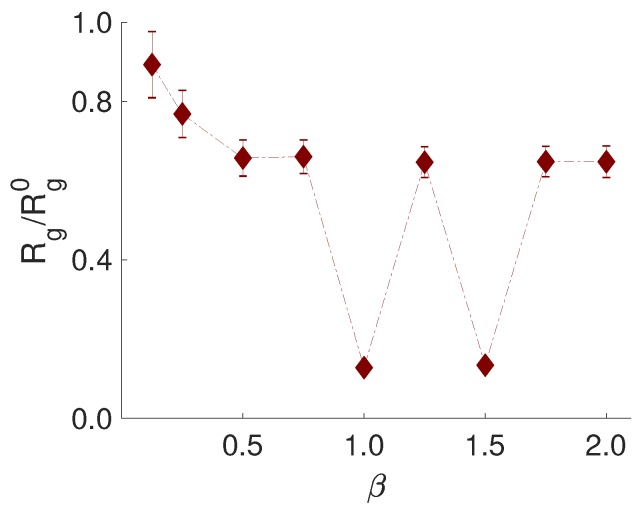
The normalized radius of gyration, $R_{g}/R_{g}^{0}$, of PE as a function of the stoichiometric charge-ratio, β. Error bars are included, and for some data points, the error is smaller than the symbol size. The dashed-dotted line is a guide for the eye.

The conformation of the PE was analyzed by the normalized radius of gyration, $R_{g}/R_{g}^{0}$, i.e., $R_{g}$ of the PE in the system with NPs divided by $R_{g}$ of the undisturbed chain. From Figure 5, it is shown that the PE in the complex was more contracted than the undisturbed PE and that $R_{g}/R_{g}^{0}$ exhibited two pronounced minima for β= 1.0 and 1.5, where the Kratky plots indicated a compact shape of the obtained complexes.

**Figure 6:**
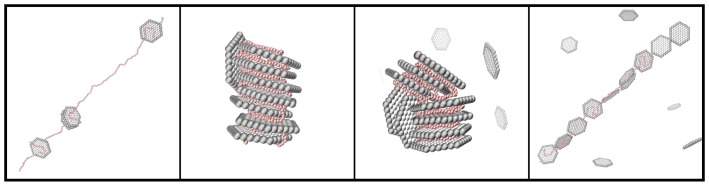
Representative snapshots of the different structures with β= 0.5, 1.0, 1.5, and 2.0 (from left to right). The counterions are omitted for clarity; the NPs are shown in grey; and the PE is shown in red.

From the simulations, representative snapshots of the systems were extracted (see Figure 6), where two different structures of the complex depending on β were found, i.e., a band-like formation, as well as a stack of NPs, the latter for β= 1.0 and 1.5. These snapshots validated the above mentioned results regarding the shape and conformation of the PE within the complex and concluded that the state diagram of the obtained complexes was clearly depending on β.

### 3.2. Effect of PE Length

**Figure 7:**
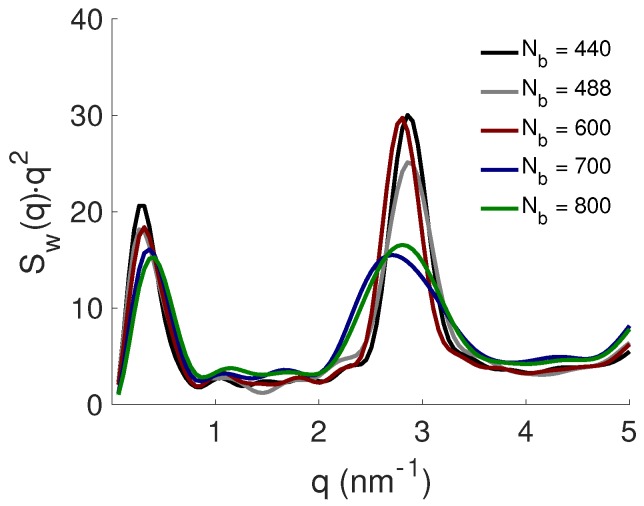
The NP-NP structure factor, $S_{w}\left(q\right)$, presented as Kratky plots for the effect of PE length.

The effect of the PE properties on the complexation between the PE and the NPs was studied; see the system S.II in Table 1 for specifications. Here, the number of the latter was constant, $N_{p}=8$, whereas the length of the former was varied between $N_{b}=$ 440–800. As expected, all NPs were complexed to the PE, and the reader should notice that when the PE length was increasing, the degree of undercharging was also increasing, i.e., there was an excess of PE charges in the system. The Kratky plot between the NPs in Figure 7 shows that the formed complexes attained a compact shape independently of the number of beads within the PE, although there was a linear decrease in the intensity as is visible by the peak positions at low *q*, indicating that the periodicity between the NPs decreased. This was further confirmed by the Bragg peaks at high *q*, where its position shifted towards lower *q*-values with an increase in PE length, i.e., the distance between the NPs was increasing; see Table 2.

**Figure 8:**
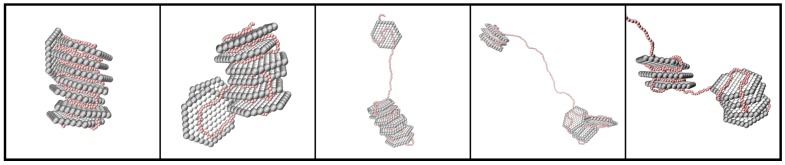
Representative snapshots of the complex formed for the PE with $N_{b}=$ 440, 488, 600, 700, and 800 (from left to right). The counterions are omitted for clarity; the NPs are shown in grey; and the PE is shown in red.

Furthermore, the normalized radius of gyration $R_{g}/R_{g}^{0}$ increased with $N_{b}$, and the representative snapshots in Figure 8 show that the NPs were forming smaller distinct clusters along the PE in a face-to-face configuration when $N_{b}$ was increasing, i.e., $\beta^{c}<1$. Hence, the free energy of the system was decreasing due to the balance between the increase in conformational entropy of the PE and the enhanced electrostatic attractive energy between the NPs and the PE.

The effect of the number of NPs was also investigated. An excess of NPs displayed a linear increase in the average number of NPs complexed to the PE, although $\beta^{c}$ and $R_{g}/R_{g}^{0}$ converged to 1.1 and 0.1, respectively (data not shown).

### 3.3. Effect of the PE Total Charge

**Figure 9:**
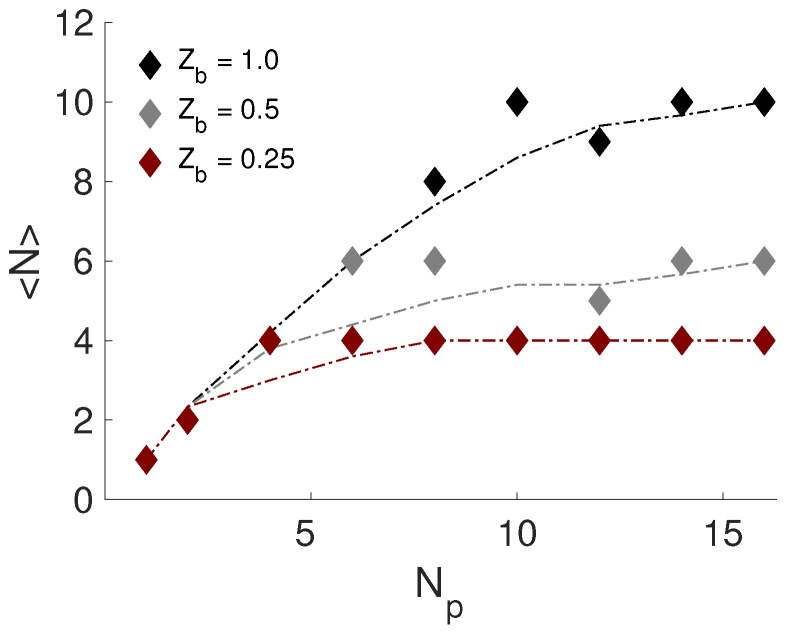
The average number of NPs complexed to the PE, 〈N〉, as a function of the number of NPs, $N_{p}$, added to the system. The dashed-dotted lines are an implemented smooth function.

The effect of the linear charge density, and thereby the total charge of the PE, defined as $Z_{\mathrm{chain}}=N_{b}\cdot Z_{b}$, was altered in the system S.III, whilst keeping the length of the PE constant. Figure 9 show that 〈N〉 was decreasing with respect to the decrease of the total charge of the PE, although the possibility of overcharging was increasing, as displayed in Table 3 and in Figure S2 in the Supplementary Materials. For example, comparison of β=2.0 resulted in $\beta^{c}$ of 1.25, 1.5, and 2.0 for $Z_{b}$ of 1.0, 0.5, and 0.25. Notice that this was independent of the linear charge density of the PE, where the maximum number of complexed NPs was reached; cf. plateau-values.

**Figure 10:**
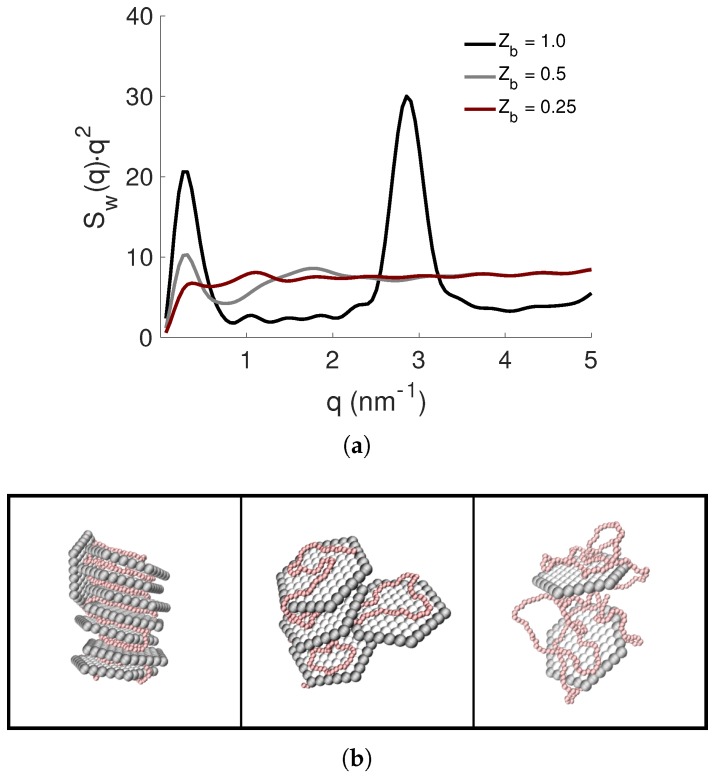
(**a**) The NP-NP structure factors, $S_{w}\left(q\right)$, presented as Kratky plots, and (**b**) representative snapshots of the structures with $Z_{b}=$ 1.0, 0.5, and 0.25 (from left to right) for $\beta^{c}=1.0$. The counterions are omitted for clarity; the NPs are shown in grey; and the PE is shown in red.

From the NP-NP structure factors, $S_{w}\left(q\right)$, presented as Kratky plots in Figure 10a, it was observed that the shape of the complexes were dependent on the total charge of the PE and that there was a transition from a compact to more elongated shape when $Z_{b}$ was decreasing. Furthermore, the Bragg peak was only visible for $Z_{b}=1.0$, implying that the face-to-face configuration of the NPs was replaced by a looser structure. These results correlated with the fact that the PE became more contracted with a decrease in the total charge of the PE for $\beta^{c}=1.0$ (see the normalized radius of gyration $R_{g}/R_{g}^{0}$ in Table 3), which probably was an effect of an increase in its conformational entropy. The above given results correlated well with the snapshots presented in Figure 10b, and in Figure S3 in the Supplementary Materials.

### 3.4. Effect of PE Flexibility

**Figure 11:**
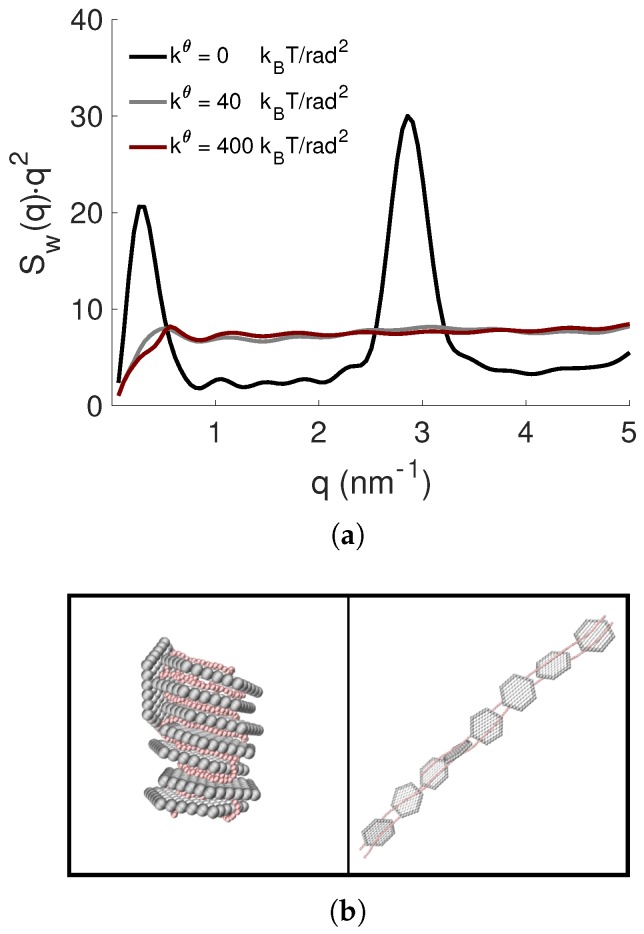
(**a**) The NP-NP structure factors, $S_{w}\left(q\right)$, presented as Kratky plots, and (**b**) representative snapshots of the complexes with $k^{\theta }=$ 0, and 400 $k_{B}T/{\mathrm{rad}}^{2}$ (from left to right) for $N_{p}=8$. The counterions are omitted for clarity; the NPs and the PE are shown in grey and red, respectively.

The effect of the flexibility of the PE on the formed complexes was investigated by varying the strength of the bead-bead angular potential in Equation (7) of the PE, refereed to as the system S.IV, while keeping the PE length constant. The angular force constant $k^{\theta }$ was set to 0, 40, and 400 $k_{B}T/{\mathrm{rad}}^{2}$, in order to mimic a flexible, a semiflexible, and a stiff chain. Here, both the average number of complexed NPs, as well as the degree of overcharging were within statistical uncertainties, equal independent of PE flexibility; see Figure S4 and Table S1 in the Supplementary Materials. The Kratky plots in Figure 11a for $N_{p}=8$, i.e., $\beta^{c}=$ 1.0, show that the complex became less compact when the PE flexibility was decreasing, which was also confirmed by the diminishing Bragg peak. The normalized radius of gyration showed that the conformation of the PE was remarkably different among the complexes, where it became 0.13, 0.60, and 1.03 with increasing stiffness. Notice though that within the complex where the stiff PE resided, it became even more elongated than for the undisturbed PE in the bulk. These trends are confirmed by the representative snapshots in Figure 11b, and in Figure S5 in the Supplementary Materials.

**Figure 12:**
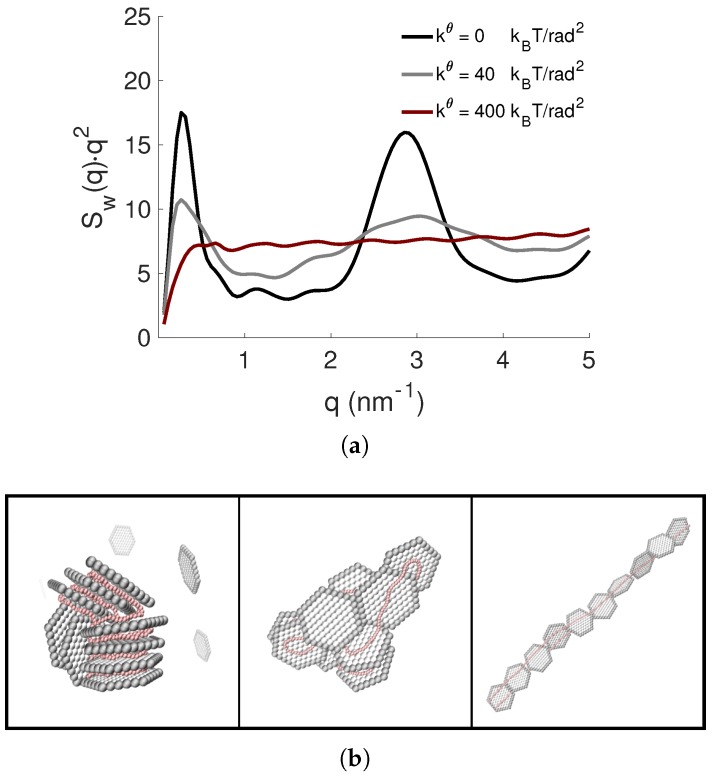
(**a**) The NP-NP structure factors, $S_{w}\left(q\right)$, presented as Kratky plots, and (**b**) representative snapshots of the complexes with $k^{\theta }=$ 0, 40, and 400 $k_{B}T/{\mathrm{rad}}^{2}$ (from left to right) for $N_{p}=12$. The counterions are omitted for clarity; the NPs and the PE are shown in grey and red, respectively.

The PE flexibility can give rise to a rich state diagram with respect to $\beta^{c}$, for example for $N_{p}=12$, i.e., when the PE was slightly overcharged by NP charges, a Bragg peak for the semiflexible PE began to be discerned, and generally, the repulsive interactions among the NPs in the complex gave rise to a looser structure. These features are shown in the Kratky plots and the representative snapshots in Figure 12. The position of the Bragg peak for the semiflexible PE corresponded to a *d*-spacing of ∼2.1 nm, which was smaller than for the flexible one with $d_{\mathrm{Bragg}}=2.3$ nm, originated from a stronger adsorption of the PE to the NPs. Moreover, the periodicity between the latter was reduced when increasing the stiffness since the PE possessed a lower chain conformational entropy.

### 3.5. Effect of NP Charge and Rim

Generally, the interaction between the PE and the NP was governed by electrostatic interactions, where the driving force of complexation was due to the gain in entropy upon counterion release, and the structure of the formed complex depended on the electrostatic attractive interaction between the PE and the NPs in combination with the PE conformational entropy. The NP had a negatively charged face and a positively charged rim, where the latter corresponded to approximately 10% of the former. Here, we studied the impact of the rim charge by comparing three different systems: (a) the PE with $N_{b}=440$ where the NP had a net negative charge of $Z_{p}=-55$, i.e., both face and rim charges were taken into consideration; (b) the length of the PE was increased to $N_{b}=488$, and thus, it neutralized only the face charges of a total of eight NPs, although the rim charge was still present; whereas in (c), the same system as in (b) was evaluated with the exception that the rim charge of the NP was set to zero.

**Figure 13:**
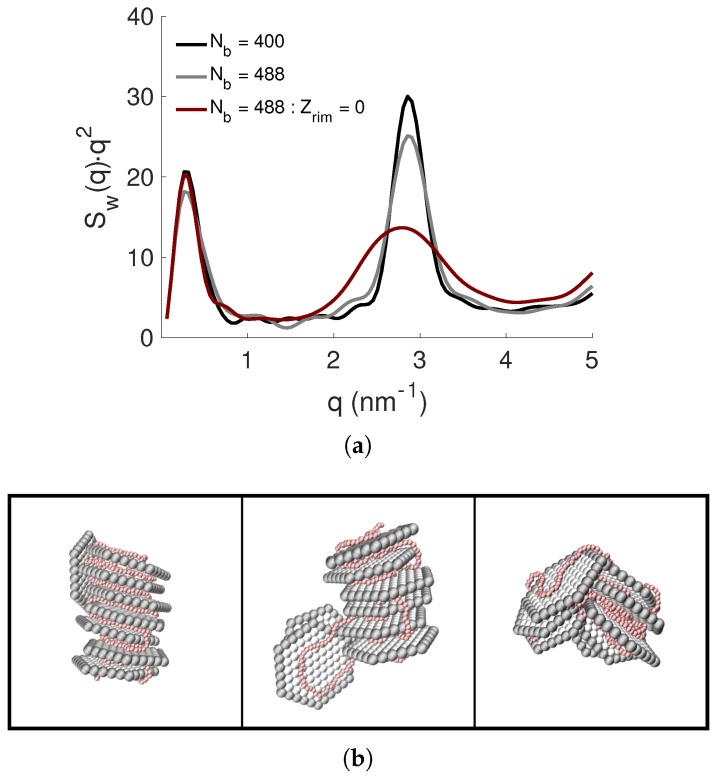
(**a**) The NP-NP structure factors, $S_{w}\left(q\right)$, presented as Kratky plots, and (**b**) representative snapshots of the different structures with the PE length of $N_{b}=$ 440, 488, and 488 with $Z_{\mathrm{rim}}=0$ (from left to right) for $\beta^{c}=1.0$. The counterions are omitted for clarity; the NPs and the PE are shown in grey and in red, respectively.

There were no larger discrepancies regarding the average number of complexed NPs between the different systems (see Figure S6 and Table S2 in the Supplementary Materials); nor in the shape of the complexes, which were compact; see the Kratky plots obtained from the NP-NP structure factor in Figure 13a. The main effect is shown at higher *q*-values where the most pronounced Bragg peak was observed when there was a charge-matching between the PE charge and the net charge of the NPs. The Bragg peak became less affected if there was charge-matching between the PE and the face of the NP where the rim charge was intact. By eliminating the rim, the intensity of the Bragg peak decreased noticeably, where also the width of the peak became broader. The positions of the Bragg peaks were consistent throughout ($d_{\mathrm{Bragg}}=2.2$ nm), indicating that the type of charge-matching within the system, or that the type of rim charge of the NPs, did not affect the adsorption of the PE to the NPs; instead, the main effect of the rim charge was visible in the shape, the structure, as well as in the configuration of the formed complex, which is clearly shown in the state diagram in Figure 13b, and in Figure S7 in the Supplementary Materials. Here, the periodicity of the NPs within the complex was reduced when eliminating the rim charges, resulting in a less ordered structure of stacked NPs with both face-to-face and face-to-edge configurations.

**Figure 14:**
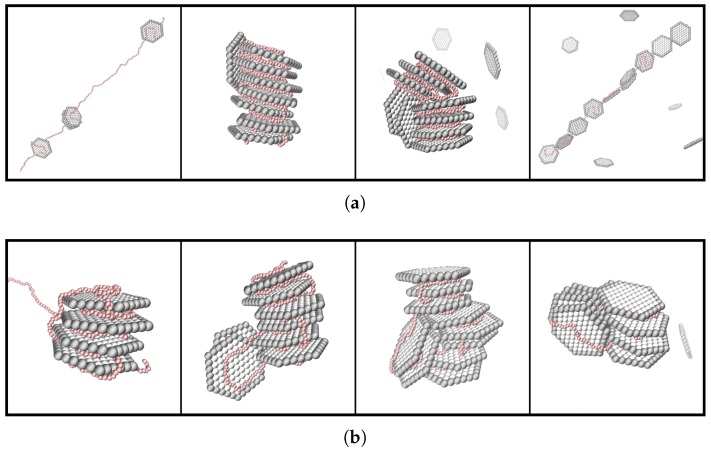
Representative snapshots of the different structures with β= 0.5, 1.0, 1.5, and 2.0 (from left to right) for (**a**) $N_{b}=440$, and (**b**) $N_{b}=488$. The counterions are omitted for clarity; the NPs and the PE are shown in grey and in red, respectively.

Furthermore, by considering the type of charge-matching between the PE and the NPs in the system, different state diagrams were obtained, illustrated in Figure 14, as an effect of the systems’ charge-ratio. For the system with charge-matching between the PE charge and the net charge of the NPs, the state diagram contained two different shapes of the complexes, an extended band-like formation, as well as a compact stack of NPs; while for the system where the PE was charge-matched with the face charges of the NPs, only the compact structure was obtained, with a variety of different configurations.

## 4. Conclusions

The complexation between one PE and anisotropically charged NPs was studied as a function of the number of NPs, as well as the PE properties. It was found that the structure, the shape, and the configuration of the formed complex depended on both the NP-PE charge-ratio in the system, as well as on the PE properties. For the PE that was charge-matched with the total charge of the NP, i.e., $N_{b}=440$, the PE conformation depended strongly on the charge-ratio, β, in the system, where the most contracted PE was found at β= 1.0 and 1.5. Further, depending on β, the complex can display both extended and compact shapes. For the PE that was charge-matched with the face of the NPs, i.e., $N_{b}=488$, the contraction of the PE decreased until the net charge of the complex was zero, i.e., β=1.0. For β>1.0, the PE conformation was unaffected by the number of complexed NPs, where it possessed a compact shape, consisting of stacked NPs. Considering the PE properties, an increase in the length resulted in an extension of the PE, and a less tightly bound complex was formed, whereas the shape of the complex was unaffected, resulting in stacked NPs. By reducing the total charge of the PE through the linear charge density, it became more contracted, while the complex obtained a less tightly bound structure. Upon decreasing the flexibility of the PE, a reduction in contraction was observed, where the complex was mainly in the extended band-like configuration.

Furthermore, the complex formation was also assumed to depend on the NPs’ properties; however, in this study, the model remained constant in order to reduce the number of variable parameters. By increasing the NPs’ size, the attractive interactions between the PE and the NPs are enhanced, since the interactions in the system are to a first approximation proportional to the area of the latter.

The information gained in this study concludes that the complexation between PEs and NPs could result in a versatile and rich state diagram, which indeed is difficult to predict, and dependent on the properties of the PE and the details of the model used. Due to the fact that the NPs have an anisotropic charge, and that both the PE and the NPs are highly charged, the systems might get stuck in semi-equilibrium, which makes possible structures and configurations even more difficult to foresee. This was also observed by simulating replicates of the systems to ensure convergence and avoid non-equilibrium structures. Nevertheless, with our simulation model, it was possible to tune both the characteristics of the PE and the NPs, and thus, we propose that the present model can be an useful tool to achieve a hint and an understanding of the PE-NP complexation.

## Figures and Tables

**Table 1:** Specifications of the systems.

System	$N_{p}$	$Z_{\mathrm{face}}$	$Z_{\mathrm{rim}}$	$N_{\mathrm{chains}}$	$N_{b}$	$Z_{b}$	$k^{\theta }$ ($k_{B}T$/rad${}^{2}$)
S.I	1–16	−61	6	1	440	1	0
S.II	8	−61	6	1	440–800	1	0
S.III	1–16	−61	6	1	440	0.25–1	0
S.IV	1–16	−61	6	1	440	1	0–400
S.V	1–16	−61	6	1	488	1	0
S.VI	1–16	−61	0	1	488	1	0

**Table 2:** Number of PE beads, $N_{b}$, stoichiometric charge-ratio in the system, β, complexed charge-ratio, $\beta^{c}$, average number of NPs complexed to the PE, 〈N〉, normalized radii of gyration, $R_{g}/R_{g}^{0}$, and the NP-NP *d*-spacing, $d_{\mathrm{Bragg}}$, for the effect of the PE length.

$N_{b}$	β	$\beta^{c}$	〈N〉	$R_{g}/R_{g}^{0}$	$d_{\mathrm{Bragg}}$ (nm)
440	1.00	1.00	8	0.13	2.21
488	0.90	0.90	8	0.14	2.22
600	0.73	0.73	8	0.19	2.27
700	0.63	0.63	8	0.37	2.41
800	0.55	0.55	8	0.60	2.34

**Table 3:** Number of NPs, $N_{p}$, charge of each PE bead, $Z_{b}$, stoichiometric charge-ratio in the system, β, complexed charge-ratio, $\beta^{c}$, and normalized radii of gyration, $R_{g}/R_{g}^{0}$, for the effect of the PE total charge.

$N_{p}$	$Z_{b}$	β	$\beta^{c}$	$R_{g}/R_{g}^{0}$	$Z_{b}$	β	$\beta^{c}$	$R_{g}/R_{g}^{0}$	$Z_{b}$	β	$\beta^{c}$	$R_{g}/R_{g}^{0}$
1	1.0	0.13	0.13	0.89	0.5	0.25	0.25	0.76	0.25	0.50	0.50	0.47
2		0.25	0.25	0.77		0.50	0.50	0.47		1.00	1.00	0.18
4		0.50	0.50	0.66		1.00	1.00	0.15		2.00	2.00	0.39
6		0.75	0.75	0.66		1.50	1.50	0.73		3.00	2.00	0.37
8		1.00	1.00	0.13		2.00	1.50	0.76		4.00	2.00	0.37
10		1.25	1.25	0.65		2.50	1.00	0.76		5.00	2.00	0.36
12		1.50	1.13	0.13		3.00	1.25	0.19		6.00	2.00	0.34
14		1.75	1.25	0.65		3.50	1.50	0.27		7.00	2.00	0.34
16		2.00	1.25	0.65		4.00	1.50	0.26		8.00	2.00	0.36

## Supplementary Materials

The following are available online at https://www.mdpi.com/1422-0067/20/24/6217/s1.

## Abbreviations

The following abbreviations are used in this manuscript:

PE	Polyelectrolyte
NP	Nanoplatelet
MD	Molecular dynamics
