# Supplementary material for: Polyelectrolyte-Nanoplatelet Complexation: Is It Possible to Predict the State Diagram?
Source: Int J Mol Sci. 2019 Dec 10;20(24):6217. doi: 10.3390/ijms20246217 (PMC6940988; doi:10.3390/ijms20246217)
Supplement: Supplementary file 1 [file ijms-20-06217-s001.pdf]

# Supplementary Materials: Polyelectrolyte-Nanoplatelet Complexation: Is it possible to predict the state diagram?

**Maria Jansson [1,*] and Marie Skepö [1,2,*]**

## 1. Composition of the PE-NP Complex

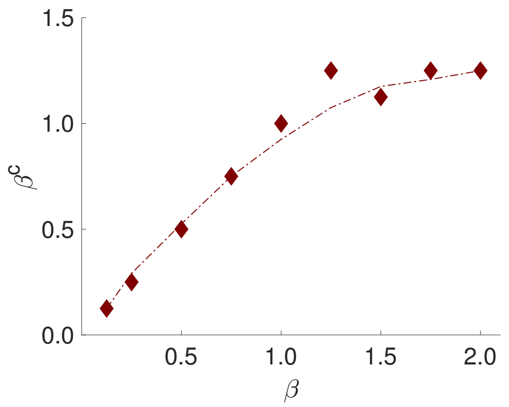


**Figure S1.** The complexed charge-ratio, $\beta^c$, as a function of the stoichiometric charge-ratio in the system, $\beta$. The dash-dotted line is an implemented smooth function.

## 2. Effect of PE Total Charge

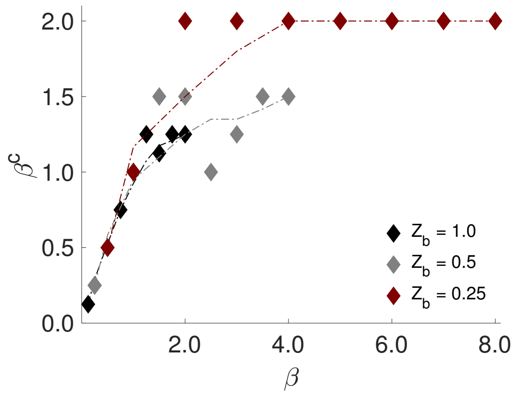


**Figure S2.** The complexed charge-ratio, $\beta^c$, as a function of the stoichiometric the charge-ratio, $\beta$, in the system. The dash-dotted lines are an implemented smooth function.

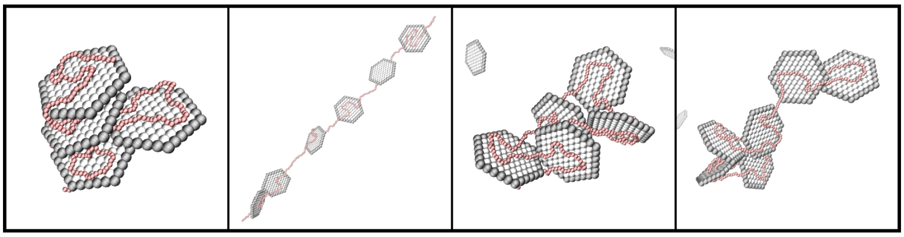

**(a)**

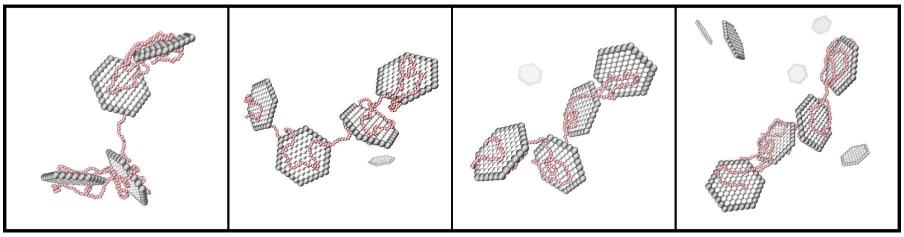

**(b)**

**Figure S3.** Representative snapshots of the structures with $\beta = 0.5$, 1.0, 1.5, and 2.0 (from left to right) for (a) $Z_b = 0.5$, and (b) $Z_b = 0.25$. The counterions are omitted for clarity, the NPs are shown in grey, and the PE is shown in red.

## 3. Effect of PE Flexibility

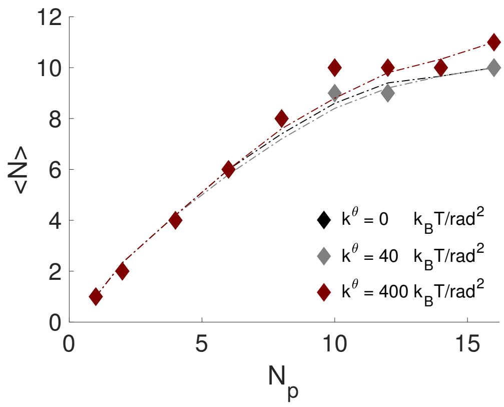


**(a)**

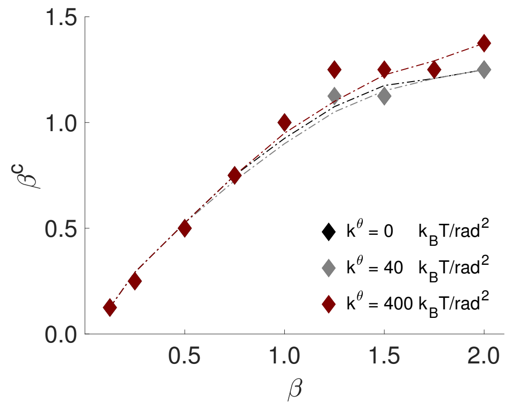


**(b)**

**Figure S4.** (a) The average number of NPs complexed to the PE, $\langle N \rangle$, as a function of the number of NPs, $N_p$. (b) The complexed charge-ratio, $\beta^c$, as a function of the stoichiometric charge-ratio, $\beta$, in the system. The dash-dotted lines are an implemented smooth function.

**Table S1.** Number of NPs, $N_\mathrm{p}$, stoichiometric charge-ratio in the system, $\beta$, angular force constant, $k^\theta$, complexed charge-ratio, $\beta^c$, average number of NPs complexed to the PE, $\langle N \rangle$, and normalised radii of gyration, $R_g/R_g^0$, for the effect of the PE flexibility. (The unit of $k^\theta$ is $k_\mathrm{B}T/\mathrm{rad}^2$).

| $N_\mathrm{p}$ | $\beta$ | $k^\theta$ | $\beta^c$ | $\langle N \rangle$ | $R_g/R_g^0$ | $k^\theta$ | $\beta^c$ | $\langle N \rangle$ | $R_g/R_g^0$ | $k^\theta$ | $\beta^c$ | $\langle N \rangle$ | $R_g/R_g^0$ |
|---|---|---|---|---|---|---|---|---|---|---|---|---|---|
| 1 | 0.13 | 0 | 0.13 | 1 | 0.89 | 40 | 0.13 | 1 | 0.94 | 400 | 0.13 | 1 | 0.92 |
| 2 | 0.25 | | 0.25 | 2 | 0.77 | | 0.25 | 2 | 0.83 | | 0.25 | 2 | 0.81 |
| 4 | 0.50 | | 0.50 | 4 | 0.66 | | 0.50 | 4 | 0.62 | | 0.50 | 4 | 0.76 |
| 6 | 0.75 | | 0.75 | 6 | 0.66 | | 0.75 | 6 | 0.61 | | 0.75 | 6 | 0.60 |
| 8 | 1.00 | | 1.00 | 8 | 0.13 | | 1.00 | 8 | 0.60 | | 1.00 | 8 | 1.03 |
| 10 | 1.25 | | 1.25 | 10 | 0.65 | | 1.13 | 9 | 0.15 | | 1.25 | 10 | 1.02 |
| 12 | 1.50 | | 1.13 | 9 | 0.13 | | 1.13 | 9 | 0.17 | | 1.25 | 10 | 1.03 |
| 14 | 1.75 | | 1.25 | 10 | 0.65 | | 1.25 | 10 | 0.61 | | 1.25 | 10 | 1.03 |
| 16 | 2.00 | | 1.25 | 10 | 0.65 | | 1.25 | 10 | 0.66 | | 1.38 | 11 | 1.01 |

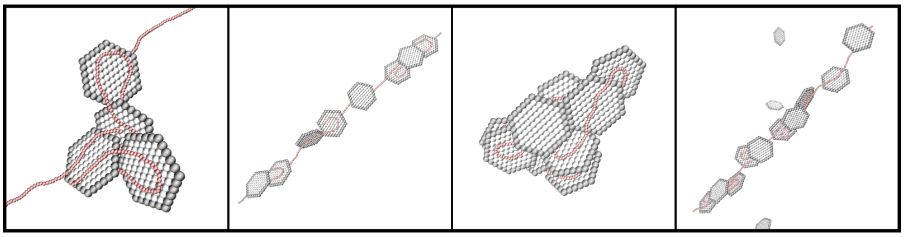

**(a)**

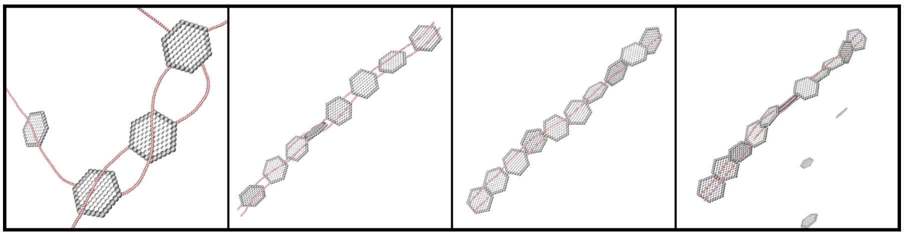

**(b)**

**Figure S5.** Representative snapshots of the structures with $\beta = 0.5$, 1.0, 1.5, and 2.0 (from left to right) for (a) $k^\theta = 40\ k_\mathrm{B}T/\mathrm{rad}^2$, and (b) $k^\theta = 400\ k_\mathrm{B}T/\mathrm{rad}^2$. The counterions are omitted for clarity, the NPs are shown in grey, and the PE is shown in red.

## 4. Effect of NP Charge and Rim

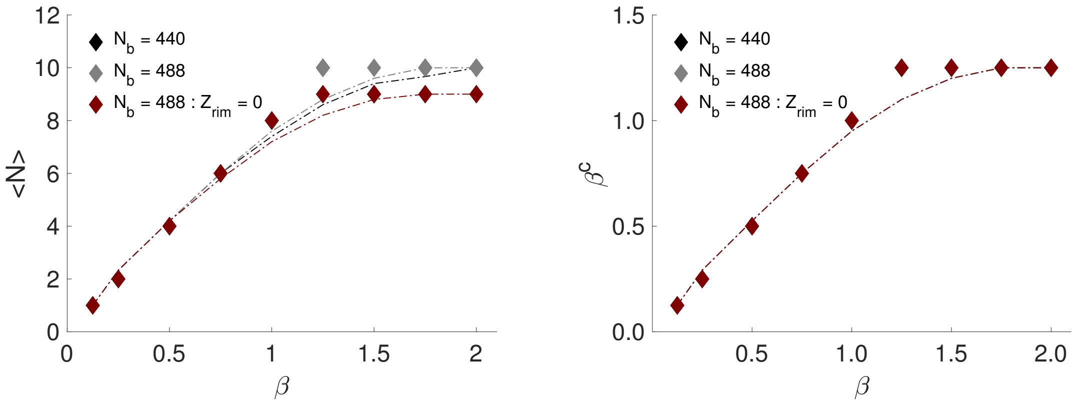


**(a)** **(b)**

**Figure S6.** (a) The average number of NPs complexed to the PE, $\langle N \rangle$, as a function of the number of NPs, $N_\mathrm{p}$. (b) The complexed charge-ratio, $\beta^c$, as a function of the stoichiometric the charge-ratio, $\beta$, in the system. The dash-dotted lines are an implemented smooth function.

**Table S2.** Number of NPs, $N_\mathrm{p}$, stoichiometric charge-ratio in the system, $\beta$, complexed charge-ratio, $\beta^c$, number of PE beads, $N_\mathrm{b}$, average number of NPs complexed to the PE, $\langle N \rangle$, and normalised radii of gyration, $R_g/R_g^0$, for the effect of the NP charge and rim.

| $N_\mathrm{p}$ | $\beta$ | $\beta^c$ | $N_\mathrm{b}$ | $\langle N \rangle$ | $R_g/R_g^0$ | $N_\mathrm{b}$ | $\langle N \rangle$ | $R_g/R_g^0$ | $N_\mathrm{b}$ | $\langle N \rangle$ | $R_g/R_g^0$ |
|---|---|---|---|---|---|---|---|---|---|---|---|
| 1 | 0.13 | 0.13 | 440 | 1 | 1.01 | 488 | 1 | 1.01 | 488 | 1 | 0.94 |
| 2 | 0.25 | 0.25 | | 2 | 0.92 | | 2 | 0.92 | with $Z_\mathrm{rim} = 0$ | 2 | 0.78 |
| 4 | 0.50 | 0.5 | | 4 | 0.53 | | 4 | 0.53 | | 4 | 0.38 |
| 6 | 0.75 | 0.75 | | 6 | 0.34 | | 6 | 0.34 | | 6 | 0.16 |
| 8 | 1.00 | 1.00 | | 8 | 0.14 | | 8 | 0.14 | | 8 | 0.12 |
| 10 | 1.25 | 1.25 | | 10 | 0.13 | | 10 | 0.13 | | 9 | 0.12 |
| 12 | 1.50 | 1.25 | | 9 | 0.13 | | 10 | 0.13 | | 9 | 0.12 |
| 14 | 1.75 | 1.25 | | 10 | 0.13 | | 10 | 0.13 | | 9 | 0.12 |
| 16 | 2.00 | 1.25 | | 10 | 0.13 | | 10 | 0.13 | | 9 | 0.13 |

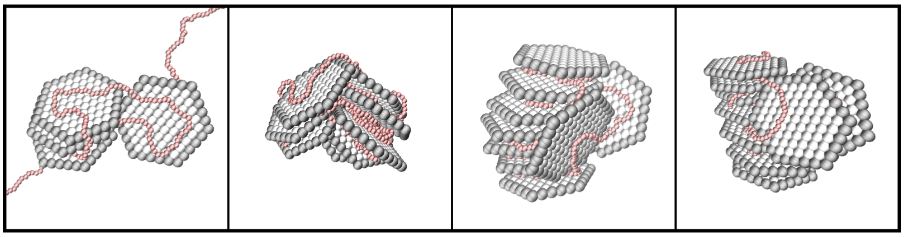

**Figure S7.** Representative snapshots of the structures with $\beta = 0.5, 1.0, 1.5$, and 2.0 (from left to right) for $N_\mathrm{b} = 488$ with $Z_\mathrm{rim} = 0$. The counterions are omitted for clarity, the NPs are shown in grey, and the PE is shown in red.
